# Specific microbiome profile in Takayasu’s arteritis and giant cell arteritis

**DOI:** 10.1038/s41598-021-84725-5

**Published:** 2021-03-15

**Authors:** Anne Claire Desbois, Dragos Ciocan, David Saadoun, Gabriel Perlemuter, Patrice Cacoub

**Affiliations:** 1 INSERM, UMR_S 959, Inflammation-Immunopathology-Biotherapy Department, Sorbonne Université, UPMC University of Paris, Paris, France; 2 Department of Internal Medicine and Clinical Immunology, AP-HP, Groupe Hospitalier Pitié-Salpêtrière, Paris, France; 3 INSERM U996, Inflammation Chemokines and Immunopathology, DHU Hépatinov, Faculté de Médecine-Univ Paris-Sud, Université Paris-Saclay, Clamart, France; 4 APHP-Hepatogastroenterology and Nutrition, Hôpital Antoine-Béclère, Clamart, France; 5 Department of Internal Medicine and Laboratory I3 “Immunology, Immunopathology, Immunotherapy” UMR 7211 (CNRS/UPMC) INSERM U959, Hôpital Pitié-Salpêtrière, 47-83 boulevard de l’Hôpital, 75013 Paris, France

**Keywords:** Cell biology, Pathogenesis

## Abstract

Recent studies have provided evidence of a close link between specific microbiota and inflammatory disorders. While the vessel wall microbiota has been recently described in large vessel vasculitis (LVV) and controls, the blood microbiome in these diseases has not been previously reported (LVV). We aimed to analyse the blood microbiome profile of LVV patients (Takayasu’s arteritis [TAK], giant cell arteritis [GCA]) and healthy blood donors (HD). We studied the blood samples of 13 patients with TAK (20 samples), 9 patients with GCA (11 samples) and 15 HD patients. We assessed the blood microbiome profile by sequencing the 16S rDNA blood bacterial DNA. We used linear discriminant analysis (LDA) coupled with linear discriminant effect size measurement (LEfSe) to investigate the differences in the blood microbiome profile between TAK and GCA patients. An increase in the levels of Clostridia, Cytophagia and Deltaproteobacteria and a decrease in Bacilli at the class level were found in TAK patients compared with HD patients (LDA > 2, *p* < 0.05). Active TAK patients had significantly lower levels of Staphylococcus compared with inactive TAK patients. Samples of GCA patients had an increased abundance of Rhodococcus and an unidentified member of the Cytophagaceae family. Microbiota of TAK compared with GCA patients was found to show higher levels of Candidatus Aquiluna and Cloacibacterium (LDA > 2; *p* < 0.05). Differences highlighted in the blood microbiome were also associated with a shift of bacterial predicted metabolic functions in TAK in comparison with HD. Similar results were also found in patients with active versus inactive TAK. In conclusion, patients with TAK were found to present a specific blood microbiome profile in comparison with healthy donors and GCA subjects. Significant changes in the blood microbiome profiles of TAK patients were associated with specific metabolic functions.

## Introduction

Large vessel vasculitis (LVV) belong to the group of systemic vasculitis and include mainly giant cell arteritis (GCA) and Takayasu’s arteritis (TAK). LVV may lead to segmental stenosis, occlusion, dilatation and/or aneurysm formation in the aorta and/or its main branches^1^. The pathogenesis of LVV is not well understood. LVV are characterized by an inflammatory infiltrate located in the arterial wall, but the mechanisms leading to such lesions remain unclear. Weyand et al. have demonstrated that inhibitory signals, by which dendritic cells provide stop signal to T cells (through PDL1 and PD1 interactions), were defective in GCA, emphasizing the regulatory importance of arterial dendritic cells in GCA pathogenesis^1^. Specific toll-like receptors (TLR) involved in pathogen-associated molecular pattern recognition have also been shown to be involved in LVV pathogenesis^2^. These data emphasize the importance of interactions between antigen-presenting cells (APC) and T cells and suggest the role of antigenic triggers promoting an uncontrolled immune response. Although interactions between pathogen agents and dysfunction of immune cells seem likely, there is currently not strong data supporting this hypothesis.

The close relationship between gut dysbiosis and altered immune response has been well established in recent studies and such alterations may be involved, at least in part, in the pathogenesis of autoimmune diseases^3^. It has long been thought that blood is a sterile environment. However, recent sequence-based studies have revealed that changes in blood microbiota are associated with various diseases^4,5^. Consistently, emerging data suggesting the presence of different communities of microbes in large-vessel diseases are recently increasing^6^. The study of blood microbiota is particularly interesting because it is a reflexion of various microbiota (gut, oral, nasal) and thus allows determination of the presence of bacteria or bacterial DNA that have crossed the mucosal barrier. Such bacteria may have direct interactions with the immune cells present within the vessels, leading to activation of the immune response.

In the present study, we aimed to evaluate the blood microbiota of patients with LVV compared with the microbiota of healthy blood donors (HD).

## Methods

Blood samples from consecutive patients with LVV who had met the criteria for GCA or TAK and from healthy blood donors were collected using dry tubes (30 mL)^7–9^. We did not use samples with sterile saline for controls. All methods were carried out in accordance with relevant guidelines and regulations.

LVV experimental protocols were approved by our institutional ethics review board (Comité de Protection des Personnes, Ile-de-France VI). Informed consent was obtained from all subjects. Disease activity was defined according to the presence of the following criteria: (1) new ischemic vascular symptoms (e.g. claudication, ischemic thoracic or abdominal pain, bruit or asymmetry in pulses, pulse abolition); (2) new LVV lesion or worsening of pre-existing lesions on imaging; (3) systemic clinical features (e.g. weight loss, fever); and (4) biological activity of disease (increased ESR and/or CRP). Disease was considered active if the score was 2 or more, and inactive in the remaining cases.

Blood was centrifuged at 4 °C immediately after sampling for 5 min at 2500 g. Serum was aliquoted into separate polypropylene tubes, which were immediately stored at − 80 °C until analysis. Bacterial DNA was extracted from 300 μL of serum from fasting specimens collected in the morning, as previously described^4,10^. The concentration of 16S rRNA gene copies normalized to 1 mL of serum in each sample was determined by real-time qPCR using primers EUBF 50-TCCTACGGGAGGCAGCAGT-30 and EUBR 50-GGACTACCAGGGTATCTAATCCTGTT-30^10,11^. As many reagents required in the qPCR and sequencing pipeline contain bacterial DNA, which can be misinterpreted as present in the samples, numerous combinations of reagents were tested to minimize bacterial contaminants. The protocol was adapted to increase the yield of amplification of the bacterial DNA present in the blood. Numerous controls were performed both in vitro and in silico to ensure the absence of artefacts (such as bacterial DNA contaminants from reagents or nonspecific amplification of eukaryotic DNA), as previously described^10^. The V3-V4 hypervariable regions of the 16S rDNA were amplified and quantified by qPCR, and sequenced with MiSeq technology (Vaiomer, Labège, France). The sequences were processed using the quantitative insights into microbial ecology (QIIME v1.9.0) pipeline, with its default parameters^12^. Sequences were then clustered into operational taxonomic units (OTUs) displaying at least 97.0% sequence similarity, using a closed reference-based picking approach in UCLUST software applied to the Greengenes 13.8 database of bacterial 16S rDNA sequences. The mean number of quality-controlled reads was 38,039 ± 3945 (mean ± SD) per sample.

After rarefaction at 30,000 reads per sample, the bacterial alpha diversity (species richness or number of taxa within a sample) was estimated based on the observed species (Faith’s PD_Whole_Tree and Shannon’s index). OTUs with a prevalence < 5% were removed from the analysis to decrease the probability of including OTUs generated by sequencing errors. The beta diversity (diversity of microbial communities between different categories) was assessed using weighted and unweighted UniFrac distances. The weighted Unifrac metric is weighted by the difference in the abundance of OTUs from each community, whereas unweighted UniFrac considers only the absence/presence of OTUs and provides different information. Both are phylogenetic beta diversity metrics. We further investigated the OTUs not identified by QIIME, using the Basic Local Alignment Search Tool (BLASTN program, BLAST version + 2.6.0) from NCBI Blast, against the NCBI 16S Microbial database^14,15^.

### Inferred metagenomics

The functional composition of the intestinal metagenome was predicted using Phylogenetic Investigation of Communities by Reconstruction of Unobserved States (PICRUSt)^13^. This is a computational approach that accurately predicts the abundance of gene families in the microbiota and thus provides information about the functional composition of the microbial community. A total of 6909 KEGG orthologs were assigned using the complete 16S sequence dataset, corresponding to 328 modules. Of these, 146 modules were assigned to metabolic pathways, and the rest of the analysis focused on the metabolic pathways. Circulating microbiome sequencing data is available from the Sequence Read Archive (SRA) with accession number PRJNA661427^14,15^.

### Statistical analysis

The results are expressed as means ± SEM. Alpha diversity comparisons were performed with nonparametric Student’s t-tests and Monte Carlo permutations in QIIME. Individual comparisons were performed at all levels of classification or taxonomic rank (phylum, class, order, family and genus). Taxa were compared using Mann–Whitney U tests, and the ANOSIM test with 999 permutations was used to compare distance matrices (weighted and unweighted UNIFRAC) in QIIME. The Benjamini–Hochberg false discovery rate (FDR) correction was used to correct for multiple hypothesis testing when applicable.

We used the linear discriminative analysis (LDA) effect size (LEfSe) method to identify taxa displaying the largest differences in abundance in the microbiota and predicted metagenome between groups^16^. Only taxa with an LDA score > 2 and a significance of α < 0.05, as determined in Wilcoxon signed-rank tests, are reported. The size of the circles in the cladogram plot is proportional to bacterial abundance. LEfSe and PICRUSt have been consulted online (http://huttenhower.sph.harvard.edu/galaxy/). For the remaining comparisons, we used R (v2.14.1) or GraphPad (v7.01, Graphpad Prism, Graphpad Software Inc, La Jolla, California, USA). Continuous data were compared using unpaired t-tests or Mann–Whitney U tests, as appropriate, while chi-squared tests or Fisher’s exact tests were used to compare discrete parameters. A multivariate association test with linear models (MaAsLin2) was used to find associations between clinical features (disease activity, treatment) and microbial community abundance^17^.

## Results

### Patient characteristics

A total of 13 patients with TAK (20 blood samples), 9 patients with GCA (11 blood samples) and 15 HD patients were analysed. TAK and GCA patients had mean ages of 45 (23.1; 70.6) and 74.5 (58; 84) years, and 54.5% and 85% were females, respectively (Table 1). Of the samples from TAK patients, 10 were performed when the disease was active and 10 when it was inactive; in the samples from GCA patients, 6 were active and 5 inactive. Of the TAK samples, 7 were performed without treatment at inclusion and 13 were from patients on treatments including low-dose corticosteroids (n = 2), methotrexate (n = 6), azathioprine (n = 1) or biotherapy (n = 4). Of the GCA samples, 2 were performed without treatment and the others were from patients on low-dose corticosteroids (n = 4), corticosteroids > 10 mg/day (n = 4) and tocilizumab (n = 1).

**Table 1:** Patient characteristics.

	Active GCA	Inactive GCA	Active TA	Inactive TA
	n = 6	n = 5	n = 10	n = 10
Age, med (min; max)	77.4 (58.2; 84)	70.1 (64; 80)	43.8 (23.6; 61)	41.4 (23.1; 70.6)
Gender, female, n (%)	3 (50)	3 (60)	8 (80)	9 (90)
Time from diagnosis, med (min; max)	2 (0; 5.5)	2.6 (1.5; 4.5)	2.8 (0.1; 11.6)	4.2 (0.2; 19.8)
**Treatment**				
Immunosuppressant, n (%)	1 (16.7)	0 (0)	4 (40)	4 (40)
TNF inhibitors, n (%)	0 (0)	0 (0)	2 (20)	2 (20)
IL-6 inhibitors, n (%)	0 (0)	0 (0)	0 (0)	1 (10)
Corticosteroids, n (%)	3 (50)	4 (80)	5 (50)	7 (70)
Dose of corticosteroids, med (min; max)	15 (5; 25)	11 (5; 50)	8 (5; 10)	10 (5; 30)
Pulses of corticosteroids	0 (0)	0 (0)	2 (20)	0 (0)
Antibiotics	0 (0)	1 (20)	0 (0)	0 (0)
**Symptoms**				
Ocular symptoms	3 (50)	0 (0)	1 (10)	1 (10)
Articular involvement	1 (16.7)	0 (0)	0 (0)	1 (10)
Cranial symptoms	3 (50)	1 (20)	0 (0)	1 (10)
Carotidynia	0 (0)	0 (0)	3 (30)	1 (10)
Claudication of arm	0 (0)	0 (0)	4 (40)	3 (30)
Claudication of leg	0 (0)	0 (0)	3 (30)	2 (20)
Dyspnoea	0 (0)	0 (0)	1 (10)	0 (0)
C-reactive protein (mg/l), mean (+ /− SD)	11.1 (12.8)	5.3 (6.1)	21.6 (23.6)	3 (5.1)

### Blood microbiota signature of LVV patients

**Figure 1:**
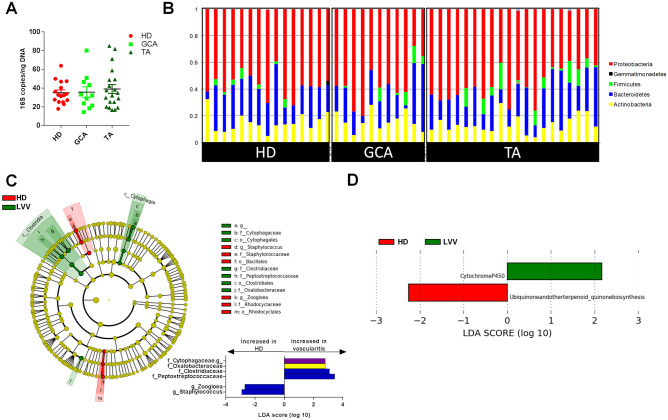
Circulating microbiome profile in patients with large vessel vasculitis (LVV). (**A**) Absolute quantity of bacterial 16 s DNA measured in the samples in the different groups. (**B**) Histograms of the circulating microbiome composition at the phyla level in HD (n = 15), GCA (n = 11) and TAK (n = 20) patients. (**C**) LDA effect size (LEfSe) cladograms showing the taxa most differentially associated with LVV (n = 26, green) or HD (n = 15, red) (Wilcoxon rank-sum test, p < 0.05). Circle sizes in the cladogram plot are proportional to bacterial abundance. The circles represent, going from the inner to outer circle: phyla, genus, class, order, and family. (**D**) KEGG pathway contributions of predicted metagenomic data in LVV and HD patients (Wilcoxon rank-sum test, *p* < 0.05) (GCA, giant cell arteritis; TA, Takayasu’s arteritis; HD, healthy blood donors). (i) Alpha diversity—degree of taxa diversity (OTU) within a sample without regard to specific organisms present. (ii) Beta diversity—degree of microbial similarity (or dissimilarity) between 2 samples taking into account specific taxa present.

There was no difference in the absolute quantity of 16 s DNA bacterial measured in the samples from the different groups (Fig. 1A). We found no difference in terms of alpha and beta diversity or in terms of the phyla composition between the groups (Fig. 1B). Using linear discriminant analysis effect size, we identified changes in the blood microbiome between LVV and HD patients at the smallest taxonomic levels. LVV patients showed an increased abundance of Cytophagia and Clostridia at the class level compared with HD patients (Fig. 1C). At the genus level, LVV showed an increase in an unidentified taxa from the Cytophagaceae family and a decrease in Zoogloea and Staphylococcus compared with HD patients (*p* < 0.05) (Fig. 1C). Concerning the predicted metagenomic functions of the blood microbiota, LVV patients showed enrichment of Cytochrome P450 pathways and a decrease in ubiquinone and other terpenoid-quinone biosynthesis pathways compared with HD patients (Fig. 1D).

### Blood microbiota signature of TAK patients

**Figure 2:**
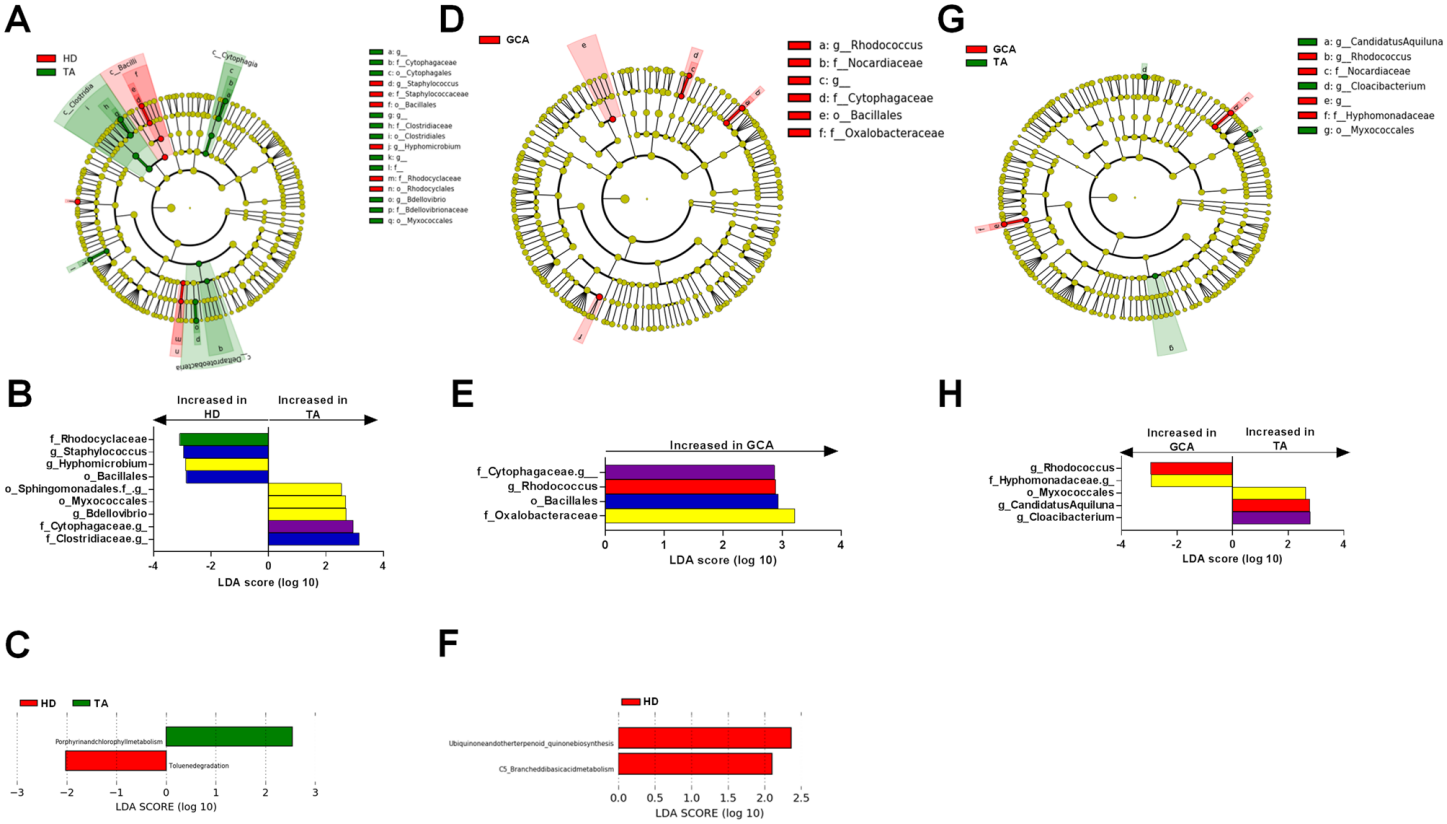
Circulating microbiome profile and predicted metagenomic function in patients with large vessel vasculitis depending on their phenotype. (**A**) LDA effect size (LEfSe) cladograms showing the taxa most differentially associated with TAK patients (n = 20) compared with HD patients (n = 15). (**B**) Specific changes in bacterial and fungal relative abundance between the two groups using LEFSe. (**C**) KEGG pathway contributions of predicted metagenomic data in TAK patients compared with HD patients. (**D**) LDA effect size (LEfSe) cladograms showing the taxa most differentially associated with GCA (n = 11) patients compared with HD patients (n = 15). (**E**) Specific changes in bacterial and fungal relative abundance between the two groups using LEFSe. (**F**) KEGG pathway contributions of predicted metagenomic data in GCA patients compared with HD patients. (**G**) LDA effect size (LEfSe) cladograms showing the taxa most differentially associated with GCA patients compared with TAK patients. (**H**) Specific changes in bacterial and fungal relative abundance between the two groups using LEFSe. LDA: Linear discriminant analysis. (*p* < 0.05). (GCA, giant cell arteritis; TA, Takayasu’s arteritis; HD, healthy blood donors).

Compared with HD, the blood microbiome of TAK patients showed an increased abundance of Clostridia, Cytophagia and Deltaproteobacteria and a decrease in Bacilli at the class level. At the genus level, there was an increase in Bdellovibrio and three unidentified taxa from the Cytophagaceae family (identified as *Pseudarcella hirudinis* using BLAST), the Clostridiaceae family (identified as *Clostridium saudiense* using BLAST) and the order Sphingomonadales (identified as *Bdellovibrio bacteriovorus* using BLAST), as well as a decrease in *Staphylococcus* and *Hyphomicrobium* (*p* < 0.05) (Fig. 2A,B). Compared with active TAK patients, inactive patients had an increased abundance of Staphylococcus. However, after adjusting for treatment using MaAsLin2, there was no difference between active and inactive patients. Concerning the predicted bacterial metagenomic functions of the blood microbiota, TAK patients showed enrichment in the porphyrin and chlorophyll pathways and a decrease in the toluene degradation pathway compared with HD (Fig. 2C). Active TAK patients showed enrichment of the porphyrin and chlorophyll pathways compared with TAK patients with inactive disease.

### Blood microbiota signature of GCA patients

Compared with HD, the blood microbiota of GCA patients showed an increased abundance of *Rhodococcus* and an unidentified member of the Cytophagaceae family (*p* < 0.05) (Figs. 2D,E). For the predicted bacterial metagenomic functions of the blood microbiota, GCA patients compared with HD showed a decrease in the ubiquinone and other terpenoid-quinone biosynthesis and the C5-Branched dibasic acid metabolism pathways (Fig. 2F). Predicted bacterial metagenomic functions were similar in TAK and GCA patients. Compared with TAK patients, the blood microbiota of GCA patients had an increase in the relative abundance of *Rhodococcus* and an unidentified member of the Hyphomonadaceae family and a decrease in *Candidatus Aquiluna* and *Coacibacterium* at the genus level (*p* < 0.05) (Fig. 2G,H).

## Discussion

Recent studies on microbiota provide evidence of a strong relationship between the microbiome and immune system regulation. The microbiome is able to induce regulatory immune responses and participate in immunological tolerance. It has now been well demonstrated that an inappropriate intestinal immune response impairs intestinal homeostasis, leading to gut dysbiosis and contributing to local and systemic inflammation and metabolic dysfunction. Altered microbiome composition has been shown to be associated with inflammatory diseases such as inflammatory intestinal disease (Crohn’s disease [CD] and ulcerative colitis [UC]), rheumatoid arthritis (RA) and systemic lupus erythematosus (SLE)^3^.

In large vessel vasculitis, such as TAK and GCA, there is no published data on the composition of the blood microbiome. To our knowledge, the present study is the first to evaluate the blood microbiome in LVV patients. Particularly in TAK patients, we found an imbalanced blood microbiota, characterized by an increased abundance of *Clostridia* (Firmicutes phylum), *Cytophagia* (Bacteroidetes phylum) and Deltaproteobacteria (Proteobacteria phylum), while we found a decrease in Bacilli (Firmicutes phylum) at the class level. Our data are consistent with an earlier recent study that found that arterial microbiome from inflammatory arteries differed from those associated with non-inflammatory aetiologies^18,19^.

However, we didn’t find any difference in the alpha diversity between LVV and HC patients. It has been suggested that a decrease in alpha diversity is linked to inflammation in different conditions^20,21^. However these observations were made in studies that analysed the faecal microbiome and not the blood microbiome. Moreover, the changes in alpha diversity are not the only link between microbiota and inflammation. The composition of the microbiota and the increase in the relative abundance of specific bacteria that could trigger a pro-inflammatory response could be an alternative hypothesis, even in the absence of decrease alpha diversity. Emerging data reporting the presence of a specific microbiome in the arteries and blood of patients with large-vessel vasculitis suggest that different communities of microbes may drive the inflammatory process in vasculitis. However, we cannot exclude to date that the presence of specific bacteria is not the consequence of the chronic inflammatory disease.

Expansion in the Proteobacteria phylum was associated with inflammatory conditions such as CD^22^. Some studies have consistently suggested that changes in the gut microbiome, especially a Proteobacteria-dominated community, predispose genetically susceptible mice to chronic colitis^22^. Taken together, these data provide evidence that expansion of Proteobacteria may trigger inflammatory responses^22^. Consistent with our findings, an increase of mice faecal Firmicutes and Proteobacteria has been reported during the autoimmune priming phase of RA^23^. A lower Firmicutes/Bacteroidetes ratio and an increased frequency of *Prevotella* and *Klebsiella* have also been described in individuals with SLE^24^. In patients with RA, a correlation between antibodies against *Porphyromonas gingivalis* and anti-citrullinated protein antibodies was found. This latter bacteria was shown to be involved in periodontal disease and able to induce citrullination through a protein containing citrulline, which is recognized by anti-citrullinated protein antibodies^25^.

Of note in autoimmune and inflammatory diseases, most data come from studies performed on gut microbiota. We found some similarities between our findings and those reported in gut microbiome in other diseases^3,22,23^. We can speculate that some bacteria or bacterial products, such as bacterial DNA, may translocate from the gut or other mucous membranes (mouth) and then interact with the immune system present within the vascular wall, leading to activation of the immune process and thus participation in LVV pathogenesis. Indeed, as a specific vascular microbiome has been shown in GCA arteries, specific changes in the blood microbiome found in our study may partly reflect an arterial microbiome^18,19^.

We observed an enrichment in the bacterial porphyrin and chlorophyll pathways in TAK patients. Chlorophyll-related compounds have been shown to have antioxidative capacities and anti-inflammatory activities^26–28^. Moreover, Lin et al. showed, in vitro, that chlorophyll-related compounds inhibit TNF-α-induced monocyte-endothelial cell adhesion, vascular cell adhesion molecule-1, intercellular adhesion molecule-1, IL-8 and NF-κB expression in human aortic smooth muscle cells^29^.These results highlight the close link between the microbiome, metabolomic changes and inflammation.

We acknowledge some limitations in the present study. The small number of patients reflects the rarity of LVV diseases. This could also explain the lack of association between disease activity and microbial composition after adjustment for potential confounding factors such as immunosuppressive therapy. One major concern is related to the origin of the bacterial DNA detected in the blood, whether from free bacteria, bacterial DNA resulting from immune degradation truly present in the blood or contamination. The significant differences found in LVV patients compared with HD patients and the high number of bacterial cells (10^6^ to 10^7^ genomes/mL) are not consistent with contamination. In the technique we used, the abundance of 16S ribosomal RNA genes is 1000-fold lower in negative controls compared with blood samples. The blood samples have also higher genus richness and a different composition, suggesting that technical contamination had no significant impact. The presence of a specific blood microbiome has already been demonstrated in several diseases such as chronic kidney diseases, liver fibrosis, diabetes mellitus or cardiovascular events^5,30,31^.

In summary, we found for the first time specific alterations of the blood microbiome in LVV patients compared with healthy donors, and between LVV types (i.e. TAK compared with GCA). These alterations were associated with enrichment of specific metabolic pathways, which may be involved in LVV pathogenesis. Some of these alterations have already been described in other inflammatory diseases and were associated with disease activity and with a potential effect on immune regulation. Further studies should include a larger group of LVV patients and assess the impact of these bacteria on activation and differentiation of T cells in LVV patients.

## References

[1] Watanabe R, Zhang H, Berry G, Goronzy JJ, Weyand CM (2017). Immune checkpoint dysfunction in large and medium vessel vasculitis. Am. J. Physiol. Heart Circ. Physiol..

[2] Pryshchep O, Ma-Krupa W, Younge BR, Goronzy JJ, Weyand CM (2008). Vessel-specific Toll-like receptor profiles in human medium and large arteries. Circulation.

[3] Nogueira AR, Shoenfeld Y (2019). Microbiome and autoimmune diseases: Cause and effect relationship. Curr. Opin. Rheumatol..

[4] Lelouvier B (2016). Changes in blood microbiota profiles associated with liver fibrosis in obese patients: A pilot analysis. Hepatology.

[5] Amar J (2019). Blood microbiota modification after myocardial infarction depends upon low-density lipoprotein cholesterol levels. J. Am. Heart Assoc..

[6] Clifford A, Hoffman GS (2015). Evidence for a vascular microbiome and its role in vessel health and disease. Curr. Opin. Rheumatol..

[7] Sharma BK, Jain S, Suri S, Numano F (1996). Diagnostic criteria for Takayasu arteritis. Int. J. Cardiol..

[8] de Souza AWS, de Carvalho JF (2014). Diagnostic and classification criteria of Takayasu arteritis. J. Autoimmun..

[9] Hunder GG (1990). The American College of Rheumatology 1990 criteria for the classification of giant cell arteritis. Arthritis Rheum..

[10] Païssé S (2016). Comprehensive description of blood microbiome from healthy donors assessed by 16S targeted metagenomic sequencing. Transfusion.

[11] Nadkarni MA, Martin FE, Jacques NA, Hunter N (2002). Determination of bacterial load by real-time PCR using a broad-range (universal) probe and primers set. Microbiology.

[12] Caporaso JG (2010). QIIME allows analysis of high-throughput community sequencing data. Nat. Methods.

[13] Langille MGI (2013). Predictive functional profiling of microbial communities using 16S rRNA marker gene sequences. Nat. Biotechnol..

[14] Kanehisa M, Sato Y, Kawashima M, Furumichi M, Tanabe M (2016). KEGG as a reference resource for gene and protein annotation. Nucleic Acids Res..

[15] Kanehisa M, Goto S (2000). KEGG: Kyoto encyclopedia of genes and genomes. Nucleic Acids Res..

[16] Segata N (2011). Metagenomic biomarker discovery and explanation. Genome Biol..

[17] Mallick H (2019). Predictive metabolomic profiling of microbial communities using amplicon or metagenomic sequences. Nat. Commun..

[18] Getz TM (2019). Microbiomes of inflammatory thoracic aortic aneurysms due to giant cell arteritis and clinically isolated aortitis differ from those of non-inflammatory aneurysms. Pathog. Immun..

[19] Hoffman GS (2019). The microbiome of temporal arteries. Pathog. Immun..

[20] Nishida A (2018). Gut microbiota in the pathogenesis of inflammatory bowel disease. Clin. J. Gastroenterol..

[21] Sheehan D, Moran C, Shanahan F (2015). The microbiota in inflammatory bowel disease. J. Gastroenterol..

[22] Shin N-R, Whon TW, Bae J-W (2015). Proteobacteria: Microbial signature of dysbiosis in gut microbiota. Trends Biotechnol..

[23] Rogier R (2017). Alteration of the intestinal microbiome characterizes preclinical inflammatory arthritis in mice and its modulation attenuates established arthritis. Sci. Rep..

[24] Hevia A (2014). Intestinal dysbiosis associated with systemic lupus erythematosus. MBio.

[25] Sakkas LI, Daoussis D, Liossis S-N, Bogdanos DP (2017). The Infectious Basis Of ACPA-positive rheumatoid arthritis. Front. Microbiol..

[26] Subramoniam A (2012). Chlorophyll revisited: Anti-inflammatory activities of chlorophyll a and inhibition of expression of TNF-α gene by the same. Inflammation.

[27] Zheng H (2018). Chlorophyllin modulates gut microbiota and inhibits intestinal inflammation to ameliorate hepatic fibrosis in mice. Front. Physiol..

[28] Hsu C-Y, Yang C-M, Chen C-M, Chao P-Y, Hu S-P (2005). Effects of chlorophyll-related compounds on hydrogen peroxide induced DNA damage within human lymphocytes. J. Agric. Food Chem..

[29] Lin K-H (2013). Chlorophyll-related compounds inhibit cell adhesion and inflammation in human aortic cells. J Med Food.

[30] Qiu J, Zhou H, Jing Y, Dong C (2019). Association between blood microbiome and type 2 diabetes mellitus: A nested case-control study. J. Clin. Lab. Anal..

[31] Shah NB (2019). Blood Microbiome profile in CKD : A pilot study. Clin. J. Am. Soc. Nephrol..

